# 2.0 Å resolution crystal structure of human polκ reveals a new catalytic function of N-clasp in DNA replication

**DOI:** 10.1038/s41598-018-33371-5

**Published:** 2018-10-11

**Authors:** Vikash Jha, Hong Ling

**Affiliations:** Department of Biochemistry, Schulich School of Medicine & Dentistry, University of Western Ontario, London, Ontario, N6A 5C1 Canada

## Abstract

Human polymerase kappa (polκ) is a distinct Y-family DNA polymerase with a unique N-terminal N-clasp domain. The N-clasp renders polκ’s high efficiency and accuracy in DNA replication and lesion bypass. How N-clasp empowers polκ in replication remains unclear due to the disordering of N-clasp. Here, we present a 2.0-Å resolution crystal structure of a polκ ternary complex with DNA and an incoming nucleotide. The structure-function study reveals an ordered N-clasp domain that brings conserved and functionally important residues in contact with the replicating basepair in the active site and contributes to the nucleotidyl transfer reaction. Particularly, a fully ordered Lys25 from the N-clasp domain is in H-bonding with the α- and γ-phosphates of the incoming nucleotide. K25A mutation reduces the polymerase activity of polκ significantly. This lysine is structurally analogous to a conserved lysine in the A-family DNA polymerases in the closed form. In contrast, Lys25 in the previous structures of polκ does not have any contacts with the incoming nucleotide, resembling an open form of a DNA polymerase. Based on structural and functional similarity, we propose a local open/closed mechanism for polκ in DNA replication catalysis, which mimics the common mechanism for all DNA polymerases.

## Introduction

Y-family DNA polymerases replicate through DNA lesions^1–4^. Humans have four Y-family polymerases, namely polη, polι, polκ, and Rev1, and each has unique DNA-damage bypass and fidelity profiles^5^. Whether the bypass will be error-free or error-prone depends on the type of lesions and the overall structural features of the enzyme. Polκ is unique in replicating accurately through N^2^-adducted guanines in the minor groove side of DNA^6–9^. Polκ is also efficient in extending from most DNA lesions^10–13^.

Polκ belongs to the DinB subfamily of Y-family DNA polymerases, which includes *E*. *coli* DinB (Pol IV) and archaeal Dpo4 proteins. Human polκ and other eukaryotic DinB homologs, however, differ from both pol IV and Dpo4 and other Y-family polymerases by the presence of an extra ~75 amino acids at their N-termini. This N-terminal extension, known as the N-clasp, is important for polκ activity and is conserved only among eukaryotic polκ proteins^14^. Deletion of the first 18 amino acids has no effect on polymerase activity, but a deletion of the first 67 residues reduces polκ’s activity^14,15^. This suggests that the region between residues 19–68 is required for complete activity of polκ. Particularly, polκ’s unique N-clasp domain supports an open conformation on the side of DNA minor grooves to accommodate bulky lesions with minor groove attachments. The N-clasp domain also stabilizes the single-stranded template downstream from the insertion site^16,17^.

To date, the available ternary complex structures of polκ are of moderate resolution (3.3–2.6 Å), and the structures of polκ available in the Protein Data Bank were crystallized in different space groups. All the structures show great flexibility in the first 31 residues in the N-terminal domain, either being completely disordered or flexible with high B-factors regardless of crystal packing environments^12,15–20^. According to the secondary-structure prediction also, the region between residues 1–32 of the polκ sequence is mostly in random coils. Lack of defined structures in the N-terminus may attribute to the resolution limitation in most of the polκ structures. In this study, we present a crystal structure of polκ containing amino acids (aa) 1–526 in complex with template-primer and an incoming nucleotide at 2.0 Å resolution. The 2.0 Å structure reveals new features that were not observed in any of the previous structures, including the well-resolved side-chain conformations in the N-clasp and finger domains and many well-defined water molecules in the active site. Most importantly, the 2.0 Å resolution structure allowed us to assign a new position for the side chain of a lysine residue (aa 25 in polκ) that interacts with the oxygen atoms of α- and γ-phosphates of the incoming nucleotide. In the previous structures of polκ, this lysine is either disordered or has different conformations which do not interact with the incoming nucleotide. Our functional analysis demonstrates that substitution of Lys25 to an Ala significantly reduces the catalytic activity of polκ, which further supports the importance of this lysine residue in polκ.

## Results and Discussion

### Overall structure

We co-crystallized polκ (aa 1–526) with DNA containing a T base at the template position and a correct incoming nucleotide dAMPNPP (dATP*). The ternary complex diffracted to 2.0-Å resolution at the synchrotron and contains two ternary complexes (Mol A and Mol B) in the asymmetric unit (Table 1). The final model of Mol A consists of polκ residues 16–224 and 282–518, DNA substrates (primer-template), an incoming dATP* and two Mg^2+^ ions. Mol B is almost identical to Mol A. The two molecules in the asymmetric unit superimpose with a root mean square deviation (r.m.s.d.) of 0.390 Å over 438 Cα atoms. However, Mol B has a more flexible N-clasp domain than that of Mol A and was traced to residue 20 only. The N-clasp in Mol B has high B-factors and disordered side chains, similar to the previously reported polκ structures. We will focus on Mol A for the new structural features in the defined N-clasp domain and describe the structure of Mol A below. Mol A and Mol B form a dimer on the palindromic DNA substrate (Fig. S1B). The formation of the dimer does not influence the overall conformation of the protein.

**Table 1:** X-ray data collection and refinement statistics.

Structure	polκ-DNA-dATP*
**Data collection**	
Space group	P2_1_2_1_2_1_
Cell dimensions	
*a*, *b*, *c* (Å)	67.6, 124.6, 165.0
Resolution (Å)^*a*^	44.1–2.00 (2.11–2.00)
R_merge_ (%)	7.0 (55.0)
I/σI	12.0 (2.8)
Completeness (%)	98.3 (97.4)
Redundancy	4.5 (4.7)
**Refinement**	
Resolution (Å)	44.1–2.00
No. reflections	90,700
R_work_/R_free_ (%)	19.9/23.4
No. atoms	
Protein	6989
DNA	898
Ligand	60
Water	484
*B*-factors (Å^2^)	
Protein	42.3
DNA	41.5
Ligand	27.1
Water	42.3
R.m.s. deviations.	
Bond lengths (Å)	0.012
Bond angles (°)	1.590

^a^Values in parentheses are for the highest resolution shell.

**Figure 1:**
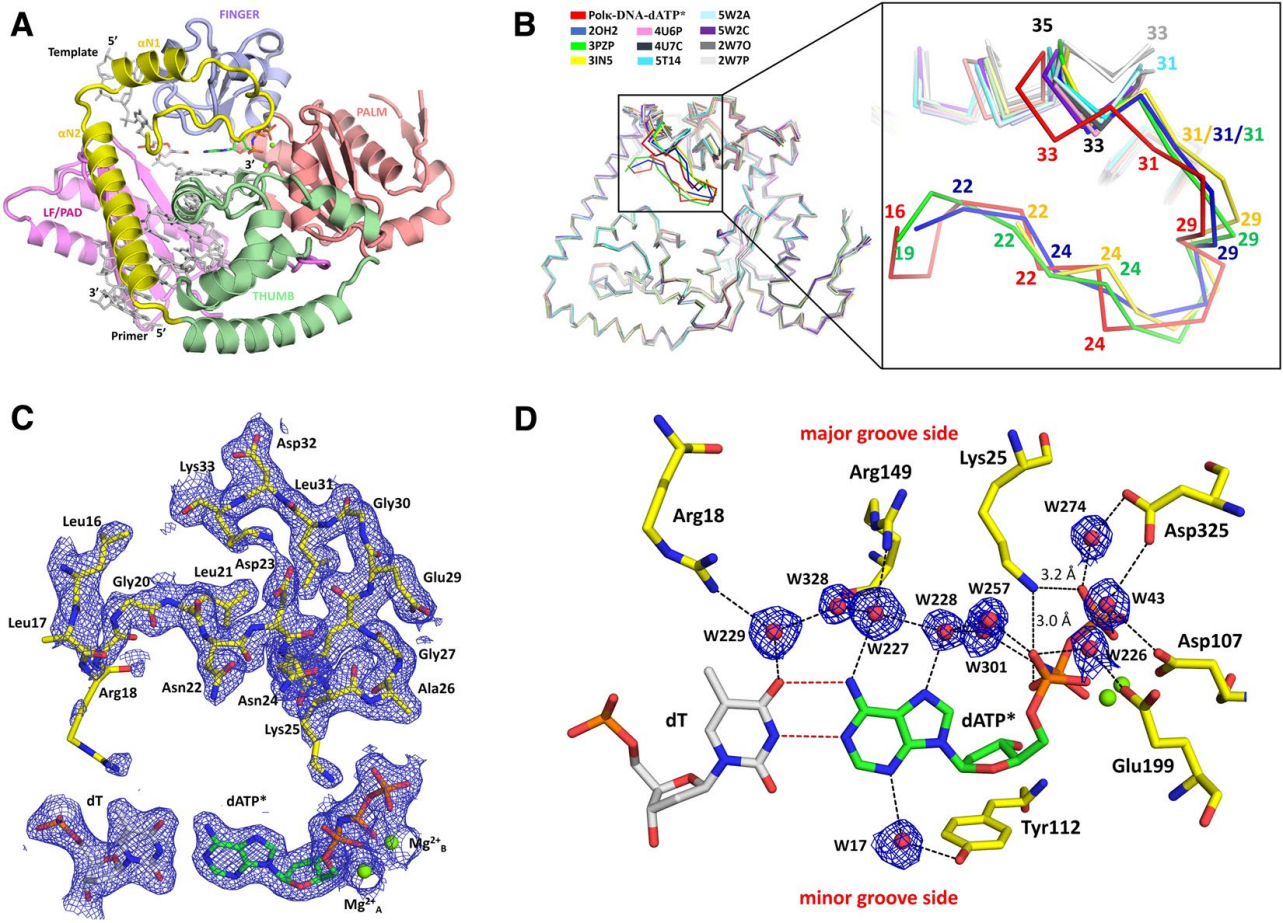
Structure of human Y-family polymerase kappa. (**A**) Cartoon diagram representing the overall structure of the ternary complex. The palm, fingers, thumb and LF/PAD domains are shown in pink, light blue, green, and magenta, respectively. The N-clasp subdomain, unique to polκ, is highlighted in yellow. DNA is represented as grey sticks, Mg^2+^ ions are shown as green spheres, and the incoming nucleotide is shown as multicolored green sticks. (**B**) Superposition of all polκ-DNA ternary complex structures showing the region of disorder in the N-terminal loop of the N-clasp. All available polκ complex structures were superposed based on their palm domains. On the left is the overall comparison of the structures. The largest deviation is observed between residues 16–35, which is highlighted in dark colors and is zoomed in on the right. The PDB IDs of the structures used for superposition are polκ-DNA-dATP* in the present study (red); 5W2A (light cyan); 5W2C (light orange); 5T14 (cyan); 4U6P (dark pink); 4U7C (light pink); 2OH2 (blue); 3PZP (green); 3IN5 (yellow); 2W7O (dark grey); and 2W7P (light grey). (**C**) 2Fo-Fc electron density map of the N-terminal residues, template dT, incoming dATP*, and two putative Mg^2+^ ions contoured at 1.0σ level. Residues are highlighted and labeled. (**D**) A close-up view of the polκ active-site region. 2Fo-Fc electron density map contoured at 1.0σ is shown for the water molecules engaged in the hydrogen-bond network with the residues and the incoming nucleotide.

The overall conformation is very similar to the previously reported polκ–DNA ternary complexes, with the N-clasp, palm, finger, thumb, and little finger domains encircling the DNA substrate (Fig. 1A). The N-terminus of N-clasp (16–26 aa) is not involved in any interactions by crystal packing (Fig. S1A), thus, the ordered conformation represents a native state in reaction. The well-ordered N-terminal residues (16–26 aa) interact with the αN1 and αN2 helices of the N-clasp, as well as finger and thumb domains, contributing to the stability of the overall structure of polκ (Fig. 1A). The N-terminal fragment also covers the DNA’s major groove and contacts the template base and incoming nucleotide in the active site (Fig. 1). New protein-DNA contacts are resolved in the 2.0 Å resolution structure (described below in detail). The N-terminal residues deviate significantly from previous structures (Fig. 1B). Comparison of all available polκ ternary complex structures reveals great flexibility in the N-terminal fragment of polκ (Fig. 1B). The majority of the structures are disordered before residue 33. Only four structures are traceable beyond residue 33, but they differ from each other in their N-termini on structural superpositions (Fig. 1B). In the 2.0 Å resolution structure, the main chains and side chains of the residues comprising the flexible loop region of the N-clasp are well defined by the electron density. The side chains of Arg18 and Lys25 are relatively flexible, as indicated by the weaker electron density and higher B factor compared to surrounding residues (Fig. 1C). The average B-factors of the flexible loop region is comparable to the average B-factor of the overall structure (50 Å^2^ for aa 16–32 vs. 39 Å^2^ for aa 16–518). This is in contrast to the previous structures, which have much higher B-factors in the N-termini than the other parts of the proteins^12,15,18^, indicating that the N-terminus is well-defined and much more ordered than those of previous structures.

The template base (dT), incoming nucleotide (dATP*), and the two Mg^2+^ ions corresponding to “metal A and B” in replicative or Y-family polymerases are well defined in the electron density (Fig. 1C). The template dT and the incoming nucleotide dATP* are in the good geometry of B-form DNA, with low tilt angles (−0.1° and 0.9°) and buckle angles (8.5° and 8.4°) in both Mol A and Mol B complexes in the asymmetric unit. The replicating base pair is in Watson-Crick base pair with two hydrogen bonds between dT and dATP* (Fig. 1D). In addition, the structure contains lots of ordered water molecules, particularly in the active site, which are engaged in an extensive network of hydrogen bonds with the incoming nucleotide, template base and the residues from the N-clasp, finger and palm domains (Fig. 1D). For example, Arg18 and Arg149 interact with the template base and incoming dATP* through W229, W328, W228 and W227 (Fig. 1D). Similarly, W274 and W43 connect Asp325 and the γ-phosphate of the incoming nucleotide by hydrogen bonds (Fig. 1D). In addition, W43, W226, and W274 also make hydrogen bonds with three acidic residues (Asp107, Glu199, and Asp325) from the palm domain, which connects them with the α- and γ-phosphates of the incoming nucleotide (Fig. 1D). Among these interactions, Arg18, Arg149, and Lys25 are the residues to be first identified in interactions with the replicating basepair in polκ by this study.

### A conserved lysine in the N-clasp is important for catalytic activity

**Figure 2:**
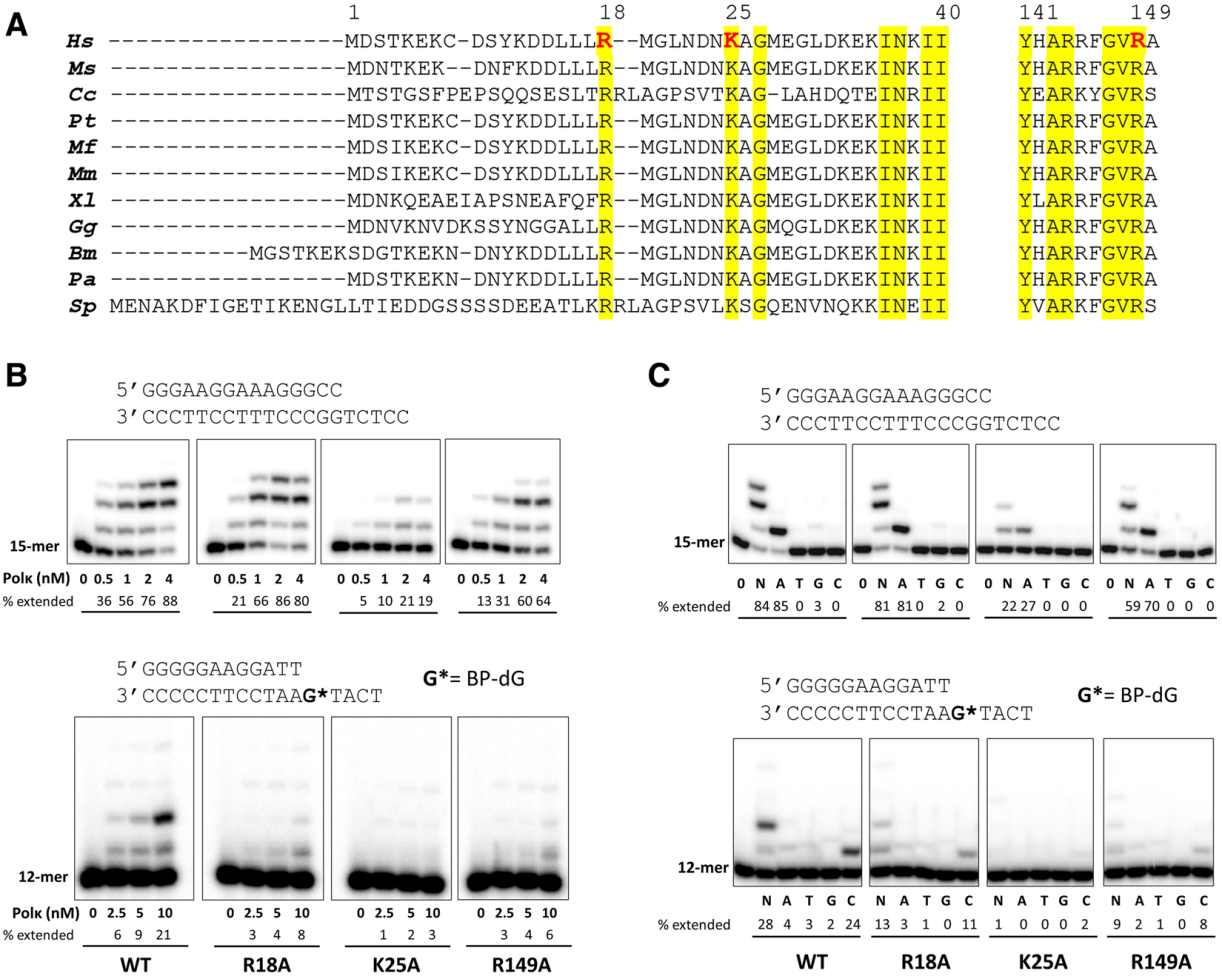
Comparison of polκ from various eukaryotes, and extension of ^32^P-labeled primers by wild-type and mutant human polκ on normal and BP-dG-containing template. (**A**) Amino acid sequence alignments of the N-terminal extension and finger domains of polκ from various eukaryotes. *Hs*: *Homo sapiens*; *Ms*: mouse (*Mus musculus*); *Cc*: *Coprinopsis cinereal*; *Pt*: *Pan troglodytes*; *Mf*: *Macaca fascicularis*; *Mm*: *Macaca mulatta*; *Xl*: *Xenopus laevis*; *Gg*: *Gallus gallus*; *Bm*: *Bos mutus*; *Pa*: *Pteropus alecto*; *Sp*: *Schizosaccharomyces pombe*. Amino acid numbering corresponds to human polκ. Conserved residues are highlighted in yellow, and polκ residues analyzed in this study are red. (**B**) Reactions were done using 50 nM DNA substrate (primer/template) with increasing concentrations of wild-type and mutant human polκ (0–4 nM for normal DNA and 0–10 nM for BP-dG-containing DNA). The ^32^P-labeled 15-mer (for normal DNA) and 12-mer (for BP-dG DNA) primer was extended in the presence of all four dNTPs for 5 minutes for normal DNA (upper panel) and 10 minutes for BP-dG-containing DNA (lower panel) at 37 °C. (**C**) Reactions were carried out with no dNTPs (0) or a single dNTP type (A, T, G, or C) or a mixture of all four dNTPs (N) using 50 nM DNA substrate and 2 nM polκ for normal DNA (upper panel) and 10 nM polκ for BP-dG-containing DNA (lower panel). The reactions times were the same as in (**B**). Cropped images in each panel (upper or lower in **B** or **C**) are a composite of lanes from the same polyacrylamide gels. Brightness and contrasts were adjusted to improve the clarity. Original (unmodified images) representing (**B**) and (**C**) are included in Supplementary Figs S4 and S5, respectively. The sequences of DNA used in the assays are shown above the respective image panels. Percentage of each primer extended in the presence of a correct dNTP or mixed four nucleotides was quantified and is indicated below each lane.

The positions of Cα atoms and the side chains of the N-terminal loop region (aa 16–32) vary significantly among the polκ structures (Figs 1B and S2). In addition, Lys25 is only found to interact with dAMPNPP in our current 2.0 Å structure and not in any other available structures of polκ. Interestingly, Lys25 in the present structure interacts with the oxygen atoms of α- and γ-phosphates of the incoming nucleotide through hydrogen bonds (Fig. 1D). Other newly observed interactions in this structure are water-mediated H-bonds between Arg18 and the template base dT and between Arg149 and the base of the incoming dATP* (Fig. 1D). The side chain of Arg149 adopts different conformations in previous structures and has no interactions with an incoming nucleotide (Fig. S3). Amino acid sequence alignment showed that Arg18, Lys25, and Arg149 are conserved among the polκ homologues in eukaryotes (Fig. 2A).

To investigate whether these conserved residues play a role in modulating polκ’s catalytic activity, we mutated these residues to alanines (R18A, K25A, and R149A) and performed DNA replication assays. Primer extension assays on the undamaged DNA substrate showed that the R18A mutant of polκ replicates DNA as efficiently as the WT polκ (Figs 2B and S4). This is consistent with the previous observations where deletion of the first 18 residues in human polκ^14,15^ and the first 22 residues in mouse polκ^21^ has no effect on the polymerase activity with normal DNA. Note that human and mouse polκ share high sequence identity, and the human and mouse deletion mutants lack the arginine at position 18 (Fig. 2A). The activity of the R149A mutant is slightly reduced in replicating DNA compared to that of WT polκ (Figs 2B and S4). Strikingly, the replication of DNA by the K25A mutant is significantly reduced compared to WT (Figs 2B and S4). Dramatic activity reduction in the mutant K25A indicates that Lys25 plays an important role in catalysis of replication. Interestingly, the activity is significantly reduced in all the mutants in the replication of 10 *S* (+)-*trans-anti*-BPDE-*N*^2^-dG- (BP-dG) adducted DNA (Figs 2B and S4), including R18A. This is consistent with the mouse polκ work done by Lui *et al*., in which the N-terminal 1–22 truncation of polκ reduces replication efficiency over 27-fold in BP-dG-DNA replication. This result indicates that Arg18 is important for BP-dG bypass, but not for normal DNA replication. Previous studies by us and others have indicated that the N-clasp is essential for the stability of the open conformation of the active site, on the minor groove side of the DNA^16,17,22^. This openness is required for polκ to accommodate the bulky adducts and bypass them. In addition to contact with the template base, Arg18 is associated with the αN2 helix of the N-clasp and contributes to the stability of the N-clasp; this, in turn, stabilizes the overall structure. The sensitivity of polκ containing R18A for BP-dG bypass reflects the importance of structural stability rendered by the N-clasp domain, as revealed in the structure.

In contrast to activity reduction, the fidelity of nucleotide incorporation opposite either an undamaged template or a template containing the BP-dG adduct was not compromised by any of these mutations. The polκ mutants predominantly incorporated the correct dNTP opposite the template, as did WT polκ (Figs 2C and S5). The primer extension data indicate that these residues do not contribute to the fidelity of polκ. In fact, the fidelity check in the high-fidelity DNA polymerases is contributed by the residues interacting with the DNA on the minor groove side. In this structure, Arg18, Lys25, and Arg149, contact the DNA substrate on the major groove side (Fig. 1D), and therefore, do not participate in fidelity checks. Our primer extension data are consistent with the structural observations.

We further analyzed K25A and R149A mutants to determine the kinetic parameters for nucleotide incorporation in DNA synthesis (Fig. S6). Our quantitative data are consistent with the observed replication profiles of these two mutants. Mutation of Lys25 or Arg149 to an alanine causes an increase in *K*_m_ (dNTP) and a large decrease in *k*_cat_ values (Table 2). The K25A mutant inserts dATP opposite dT with a rate (*k*_cat_) that is ~9-fold lower compared to WT polκ (Table 2). As a result, the catalytic efficiency (*k*_cat/_ *K*_m_) of nucleotide incorporation opposite the template relative to that for WT polκ is reduced ~20 fold (Table 2). The catalytic efficiency of the R149A mutant is also reduced ~9 fold in comparison to WT (Table 2). The kinetic study for the WT and K25A mutant in replication opposite BP-dG adduct (Fig. S6B) shows that K25A causes much more severe reduction (100-fold) in the replication efficiency of BP-dG adducted DNA than that of normal DNA (20-fold) (Table 2). The kinetic data indicate that Lys25 play an important role in bulky lesion bypass. The combination of our structural and kinetic data indicates that Lys25 and Arg149 contribute to catalysis by interacting with the incoming nucleotide.

**Table 2:** Steady-state kinetic parameters for one-base incorporation catalyzed by human wild-type and mutant polκ.

Base pairing	Polκ_1–526_	*K*_m_ (μM)	*k*_cat_ (min^−1^)	*k*_cat_/*K*_m_ (min^−1^ μM^−1^)	Efficiency relative to WT
T:dATP	WT	14.9 ± 1.2	38.1 ± 1.4	2.56	1
T:dATP	K25A	33.1 ± 2.8	4.2 ± 0.2	0.13	0.05
T:dATP	R149A	57.0 ± 4.4	18.3 ± 1.9	0.32	0.13
BP-dG:dCTP	WT	1247 ± 121	4.1 ± 0.1	3.3 × 10^−3^	1
BP-dG:dCTP	K25A	1328 ± 97	0.050 ± 0.003	3.8 × 10^−5^	0.01

### Lys25 has a function analogous to that of a conserved lysine in A-family of DNA polymerases

**Figure 3:**
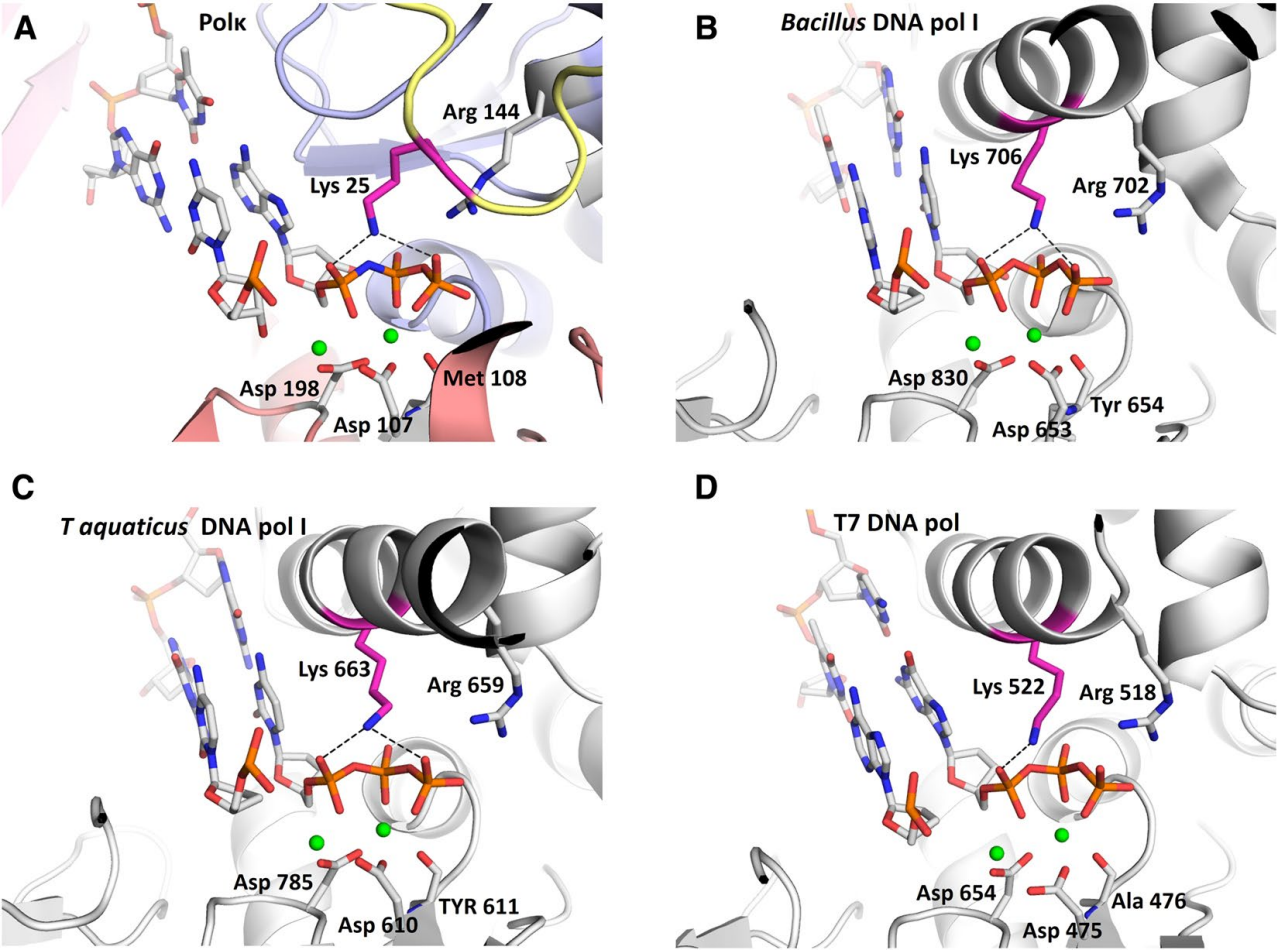
Comparison of active-site clefts of human Y-family polκ (this study) and A-family DNA pols. The structures were superposed using their incoming nucleotides, and figures were drawn on a similar scale with minor adjustments to get the best view. (**A**) polκ-DNA-dATP* ternary complex. Color codes for polymerase domains are the same as used in Fig. 1A. (**B**) DNA pol I from *Bacillus stearothermophilus* (PDB 1LV5)^30^. (**C**) DNA pol I from *Thermus aquaticus* (PDB 3KTQ)^29^. (**D**) Bacteriophage T7 DNA polymerase (PDB 1T7P)^31^. DNA in the active site of enzymes is shown as blue and grey sticks, the Mg^2+^ ions are shown as green spheres, and the residues coordinating with the Mg^2+^ ions are shown as grey and red sticks. The conserved lysine interacting with the incoming nucleotide is highlighted in hot pink, and the invariant arginine is shown as grey and blue sticks. Hydrogen bonds are indicated with black dashes.

Comparing the structures of polκ and high-fidelity DNA polymerases, we found that Lys25 is at an equivalent position to a lysine residue in the O-helix of A-family DNA polymerases (pol I) in the closed conformation (Fig. 3 and Table S1). The O-helix residues Lys706 in *Bacillus stearothermophilus* DNA pol I, Lys663 in *Thermus aquaticus* pol I, and Lys522 in T7 DNA polymerase contact the phosphates of the incoming nucleotide (Fig. 3B–D) in a similar way as Lys25 in polκ (Fig. 3A). These lysine residues are conserved in the polymerase A-family (Fig. S7). Such a conserved and structure-functionally equivalent lysine residue is Lys758 in the Klenow fragment, an A-family DNA polymerase (pol I) from *E*. *coli*.

Functionally, mutation of Lys25 to alanine (K25A) in polκ has similar effects on the kinetics parameters as the equivalent Lys 758 in the Klenow fragment. Mutant K25A causes a 9–10 fold reduction in *k*_cat_, but the affinity for the dNTP substrate (*K*_m_) is reduced only 2-fold (Table 2). In the Klenow fragment, mutation of K758A results in severe loss of polymerase activity^23–25^. Particularly, the mutation of lysine at 758 did not show a significant change in the *K*_m_, but dramatically reduced the *k*_cat_ (30–3000 fold reduction) of the polymerization reaction. Based on these results, it was suggested that Lys758 is not involved in the binding of dNTP in the first stage of the E.DNA.dNTP ternary complex formation, but may play a role in the second step leading to the conformational change of the ternary complex to the E*.DNA.dNTP complex, which is poised for catalysis^25^. The similarity of the lysine residues in structure and function indicates that the conserved lysine at 25 in polκ plays a role similar to that of the equivalent lysine residue in A-family DNA polymerases. In addition to A-family polymerases, a +1 charged residue at the similar position to K25 is also found in Y-family polymerase polη (R61)^26^, closed-form of B-family polymerase Pol II (K493)^27^, and HIV-1 reverse transcriptase (R72)^28^ (Table S1). The +1 charged residues interact with the phosphate oxygens of the incoming dNTP^26–31^ and are in the proximity of a third catalytic metal ion (metal C) observed in pol η^26,32^ and pol β^33,34^. Accordingly, the Lys 25 may also be involved in the third metal alignment which facilitates the catalysis^4,26,32^.

It is noteworthy that there is a difference between the lysine residues in A family and Lys25 in polκ. The catalytic activity of A-family polymerases largely depends on the lysine residues in the finger domains and the conformational movement of the “O” helices in which the critical lysine residues are located. Once in closed conformation, the conserved lysines are in contact with incoming dNTP for catalysis. Thus, mutation of K758A almost abolishes the activity of pol I from *E*. *coli*^23,25^. In contrast, Y-family polymerases have pre-formed active sites and no conformational changes in the finger domains upon dNTP binding is required. The open-closed switch in polκ is achieved by the conformational flexibility of N-clasp. The ordered conformation of the N-terminal fragment (closed form) likely co-exists with other disordered conformations (open forms) which is present in all previously reported pol κ structures^12,15,18^. Therefore, the overall effect of either removal of N-terminal fragment or mutation of single K25 does not have the same dramatic effect as K758A mutation in the A-family polymerase pol I^23,25^.

Conserved basic residues surrounding the phosphate moiety of the incoming nucleotide have been suggested to act as general acids to protonate the leaving pyrophosphate. The protonation promotes the release of the pyrophosphate in the nucleotidyl transfer^35,36^. The conserved lysine residues in polκ and A-family polymerases are physically close to the phosphate moiety, which makes them serve as proton donors for the leaving pyrophosphate^36^. Our and others’ function studies further support the idea that Lys25 in polκ very likely acts as a proton donor to the pyrophosphate to facilitate the leaving group, similar to A-family DNA polymerases^24,36^. The proximity of the lysine residues to the phosphate dNTP is achieved by the closed conformation of A-family polymerases and by moving the fingers domain^29,30,37^. The ordering of the N-terminus of polκ (closed form) brings Lys25 close to the dNTP, which assists pyrophosphate release and resembles the closed state. When the N-terminus of polκ undergoes local conformational changes to move Lys25 away or becomes disordered, polκ likely mimics the open form, allowing DNA translocation and for the dNTP to move in to pair with the template base.

In summary, the 2.0 Å resolution structure ordered in the N-terminus of polκ reveals new protein-DNA interactions that contribute to the catalysis of polκ in replication. The ordering of the N-clasp domain brings conserved lysine residues to contact with the replicating basepair in the active site and facilitates nucleotidyl transfer, while the internal flexibility allows DNA translocation and new incoming nucleotide binding for the next run of nucleotide incorporation. The local conformational changes in polκ reflect a similar mechanism with the open/closed conformational switches in high-fidelity DNA polymerases. The work provides an example that the essential open and closed mechanism for high fidelity DNA polymerases is also employed by a Y-family polymerase. The newly identified function of N-clasp enables polκ for efficient DNA replication, particularly for the bypass of bulky adducts generated by carcinogenic polycyclic hydrocarbons. Our work opens an avenue to study the unique domains in Y-family polymerases for their specialized functions *in vitro* and *in vivo*.

## Materials and Methods

### Preparation of wild-type (WT) and mutant polκ

Human polκ (aa 1–526) was expressed and purified according to the method described previously^16,17^. In short, the polκ gene encoding residues 1–526 was cloned into the pHIS-Parallel1 vector with hexa-histidine (6X-His) tag. The polκ mutants in this study were generated by site-directed mutagenesis using primers containing the desired mutation. Mutations were confirmed by DNA sequencing. The polκ and mutants with N-terminal 6X-His tags were overexpressed in *Escherichia coli* Rosetta (DE3) cells with 0.5 mM IPTG and purified by Ni-affinity and ion-exchange chromatography. The 6X-His tag was removed by TEV protease before ion-exchange (HiTrap SP column) chromatography.

### Crystallization of normal DNA substrate with polκ

A 22-mer self-annealing DNA substrate 5′ TACTGGTATGTAT ATACATACC-3′ was used for crystallization of the polκ ternary complex. The DNA substrate was allowed to self-anneal by incubating at 95 °C for 5 minutes and slowly cooling to room temperature (RT). Annealed DNA was mixed with purified polκ in a 0.6:1 ratio (DNA:protein) and incubated on ice for 20 minutes. Non-hydrolysable nucleotide 2′-Deoxyadenosine-5′-[(α,β)-imido]triphosphate (dAMPNPP) was then added to a final concentration of 2 mM and the mixture was further incubated at RT for 30 minutes. The ternary complex mixture was then transferred to ice before setting up the crystallization drops. Ternary complex crystals were obtained in 20–25% PEG400 and 0.2 M ammonium iodide (NH_4_I) using the hanging-drop vapor diffusion method at 22 °C.

### Data collection and structure refinement

For data collection, the crystals were picked from the drop and were soaked in 35% PEG 400, 0.2 M NH_4_I and 20% ethylene glycol before flash freezing in liquid nitrogen. X-ray diffraction data were collected at the beamline 24-ID-E operated by the Northeastern Collaborative Access Team (NE-CAT) at the Advanced Photon Source. The best crystal diffracted to 2.0-Å resolution. Data were processed and scaled using programs iMOSFLM and SCALA^38^. The crystal belonged to space group P2_1_2_1_2_1_ and contained two molecules per asymmetric unit. The structure was determined by molecular replacement with Phaser MR^39^ using the polκ ternary complex structure (PDB 4U6P)^16^ as a search model. Manual rebuilding and model corrections were accomplished using Coot^40^. Structure refinement was performed using REFMAC5^41^, and figures were prepared using the molecular graphics program PyMOL (http://www.pymol.org). The data processing and refinement statistics are listed in Table 1.

### DNA replication assays

Standard DNA polymerase reactions (10 μl) were performed in 50 mM Tris (pH 7.5) buffer containing 5 mM MgCl_2_, 250 μg/ml BSA, 5 mM DTT and 2.5% glycerol with 50 nM primer-template DNA at 37 °C. A 5′-^32^P-labeled primer was annealed with the template and used for primer extension assays. The sequences of oligonucleotides used for assays are shown in the respective figures. All reactions were initiated by the addition of dNTP solutions (prepared in the same buffer as above) to the preincubated enzyme/DNA mixtures, and the reactions were terminated with 20 μl of stop solution (20 mM EDTA, 95% formamide, 0.05% bromophenol blue, 0.05% xylene cyanol). The reaction products were resolved on a 20% PAGE containing 8 M urea, and gels were visualized using PhosphorImager.

### Steady-state kinetic analyses

A 5′-^32^P-labeled primer annealed with template was extended in the presence of increasing concentrations of a single dNTP. The sequences of oligonucleotides (unmodified and BP-dG modified) used for steady-state kinetic analyses are shown in Fig. 2B,C. To ensure single-hit polymerization conditions (less than 20% of the primer is extended), polκ concentrations, the nucleotide concentration range and time of reaction were adjusted for every experiment (See Fig. S6 for more details). The reactions were incubated at 37 °C using the standard DNA-polymerase assay described above, and the reaction products were separated by electrophoresis on a 20% polyacrylamide gel containing 8 M urea. The percentage of primers extended by the polymerase was calculated using Storm ImageQuant software. The rate of product formation (ʋ, nMmin-1) was plotted as a function of dNTP concentration, and the data were fit by a nonlinear regression curve to the Michaelis–Menten equation using GraphPad Prism software (GraphPad, San Diego, CA). *V*_max_ and *K*_m_ values were obtained from the fitted curves, and *k*_cat_ was calculated by dividing the *V*_max_ by the enzyme concentration.

## Electronic supplementary material

Dataset 1

## Data Availability

The atomic coordinates and structure factors of the polκ ternary complex have been deposited in the Protein Data Bank, www.pdb.org with ID code 6CST.
