# Supplementary material for: 2.0 Å resolution crystal structure of human polκ reveals a new catalytic function of N-clasp in DNA replication
Source: Sci Rep. 2018 Oct 11;8:15125. doi: 10.1038/s41598-018-33371-5 (PMC6181923; doi:10.1038/s41598-018-33371-5)
Supplement: Supplementary file 1 — Dataset 1 [file 41598_2018_33371_MOESM1_ESM.pdf]

2.0 Å resolution crystal structure of human polκ reveals a new catalytic function of N-clasp in DNA replication

Vikash Jha[1] and Hong Ling[1*]

1 Department of Biochemistry, Schulich School of Medicine & Dentistry, University of Western Ontario, London, Ontario, N6A 5C1, Canada

Supplementary Material

Supplementary table, figures and figure legends

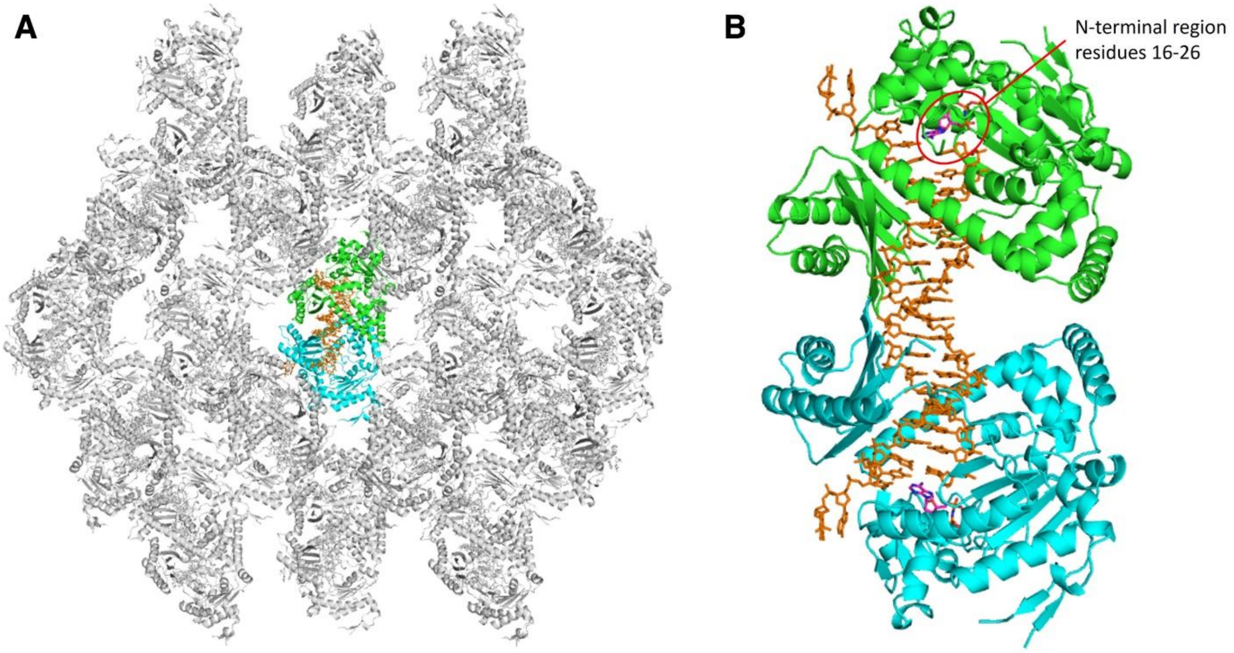

Figure S1. Crystal lattice packing and dimerization of polκ on the palindromic DNA. (A) Polκ dimers in the crystal lattice. Two polκ molecules are shown in green (Mol A) and cyan (Mol B) ribbons, palindromic DNA is shown in orange sticks, and the neighboring dimers are shown in grey. (B) polκ dimer with ~10° rotation around X and Y axis from the orientation in (A) for better view. The incoming nucleotide dAMPNPP (dATP*) in both Mol A and Mol B is shown in magenta sticks.

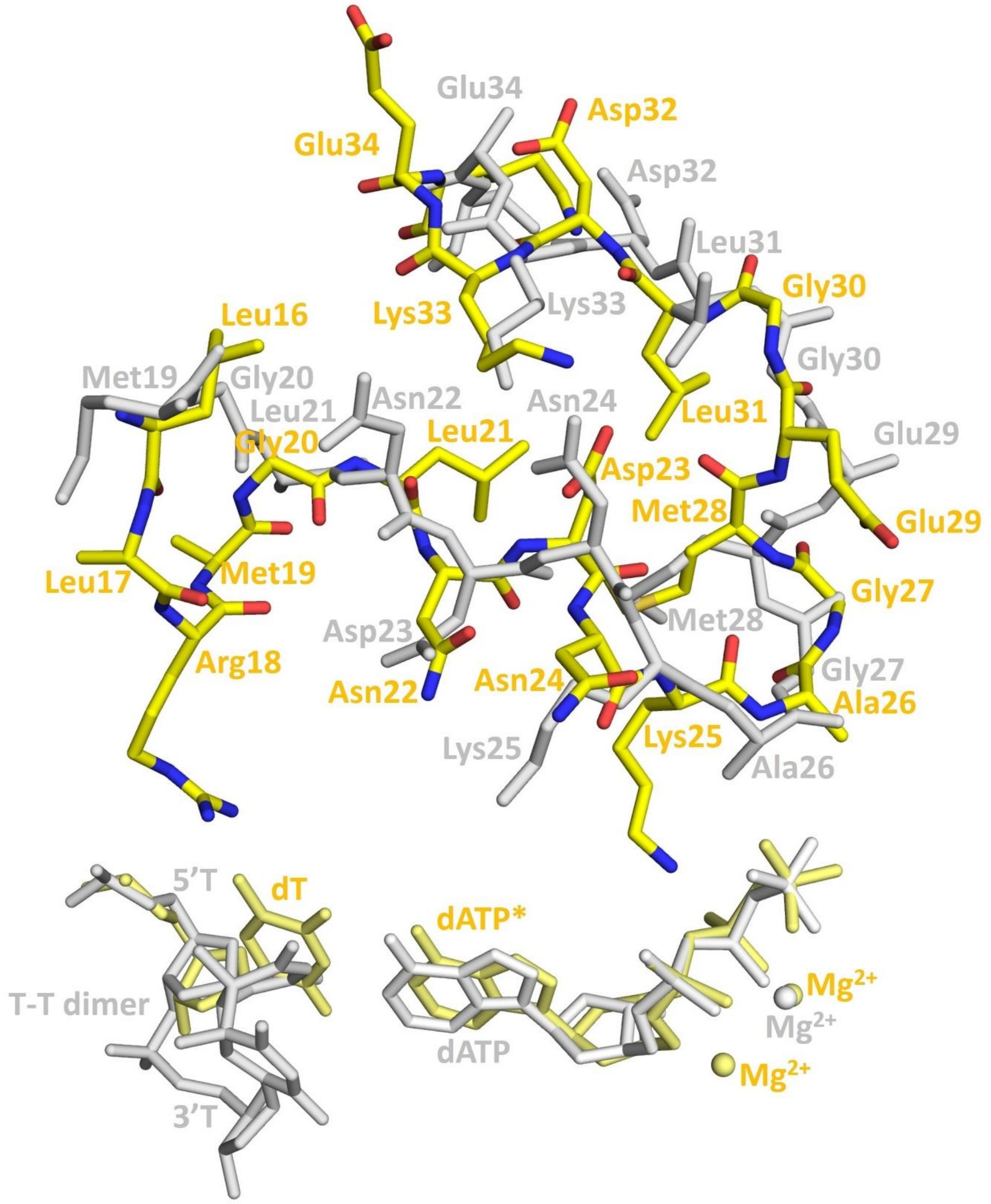

Figure S2. **Comparison of main chains and side chains of N-terminal loop residues between our high-resolution (polκ-DNA-dATP*) and a previous low-resolution structure of human polκ with a flexible N-terminal fragment (PDB 3PZP)**. The residues in polκ-DNA-dATP* are shown as yellow and multicolored sticks and are highlighted and labelled in yellow. In this

structure, the template base, incoming nucleotide and Mg^{2+} ions are labeled and highlighted in yellow. The residues, template (T-T dimer), incoming nucleotide, and Mg^{2+} ion in 3PZP are highlighted and labelled in grey. The positional shift in the main chain of residues before Met28 is evident in the two structures. This shift moves the Lys25 away from the phosphates of the incoming nucleotide in 3PZP (in grey). A similar positional shift is also observed in the other two structures of polκ that contain the extended N-terminal loop residues: PDB 2OH2 and 3IN5 (not shown here). 3PZP was used to prepare this figure, as this structure is more complete than 2OH2 or 3IN5.

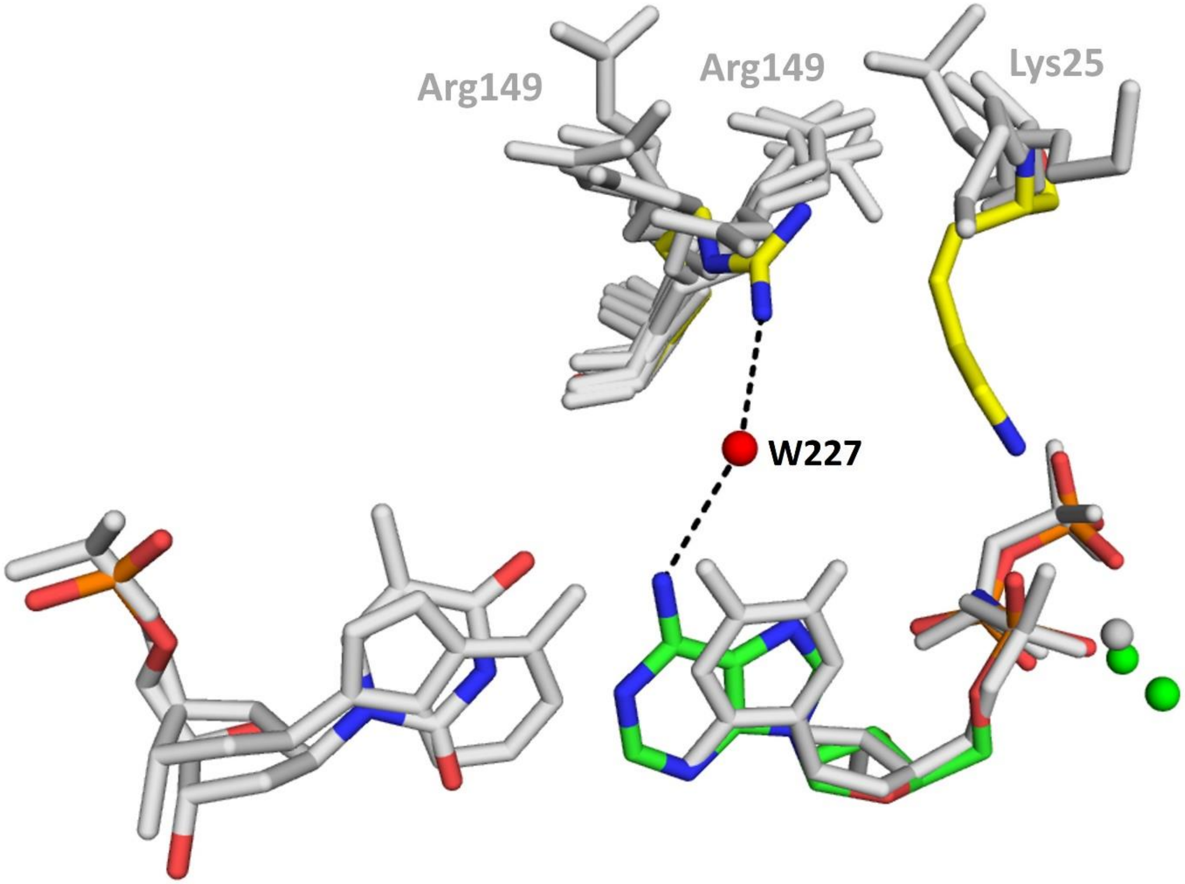

Figure S3. **Conformations of the side chains of R149 in the finger domain and K25 in the N-clasp domains of polκ ternary complex structures.** The structures used for comparison are the same as in Figure 1B. The color scheme for the present structure is the same as in Figure 1. The side chains of Arg149 and Lys25 from polκ structures are shown as grey sticks. Multiple conformations of the Arg149 side chains are labelled in grey. Incoming nucleotides, template bases and Mg^{2+} ions are only shown for the present structure and 2OH2 (in grey) for clarity.

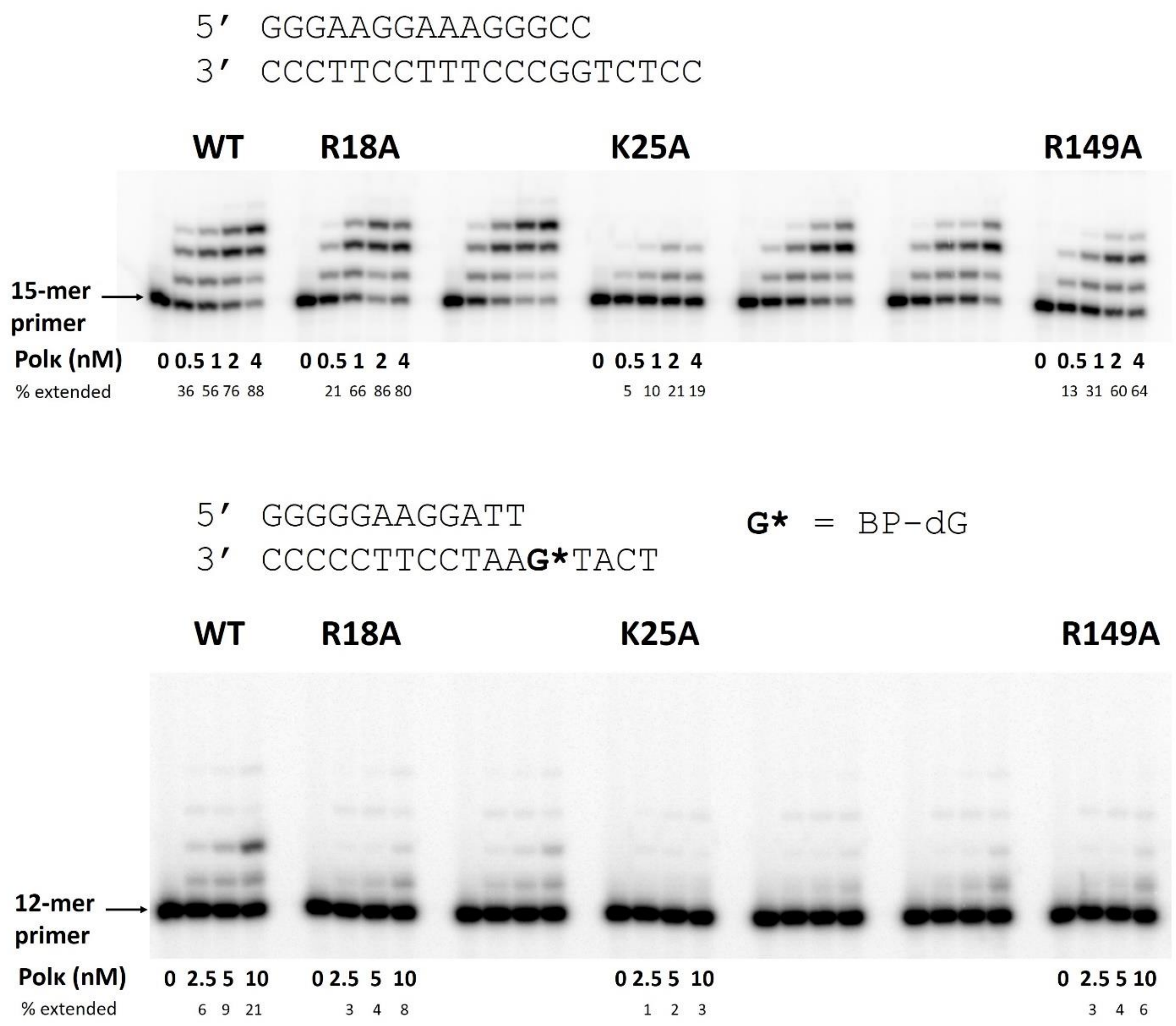

Figure S4. Full-length unmodified gel images corresponding to Figure 2B in the main text. Only the lanes which have been discussed in the main text are labelled and those which are not relevant to the study have not been labelled. See main text for details.

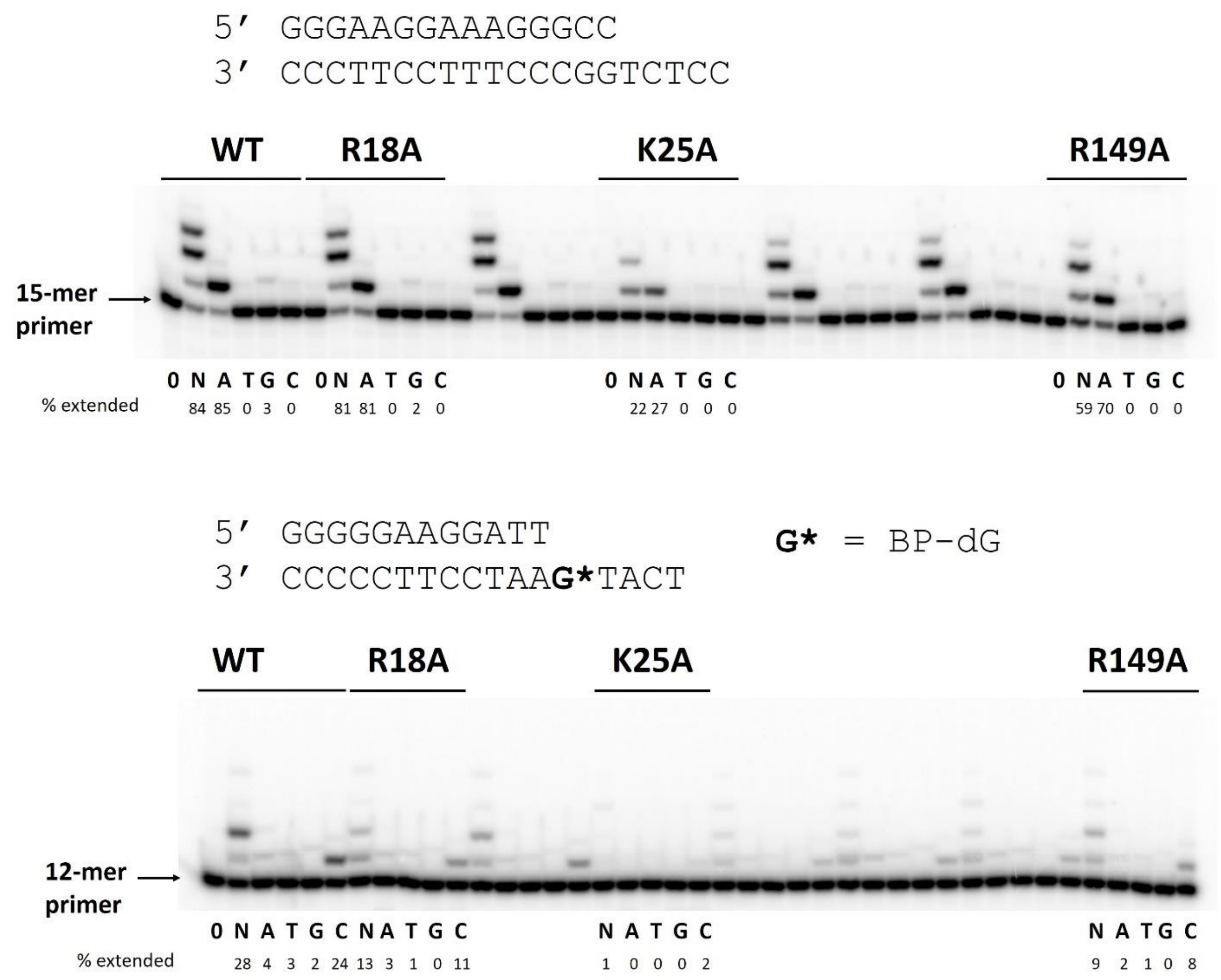

Figure S5. Full-length unmodified gel images corresponding to Figure 2C in the main text. Only the lanes which have been discussed in the main text are labelled and those which are not relevant to the study have not been labelled. See main text for details.

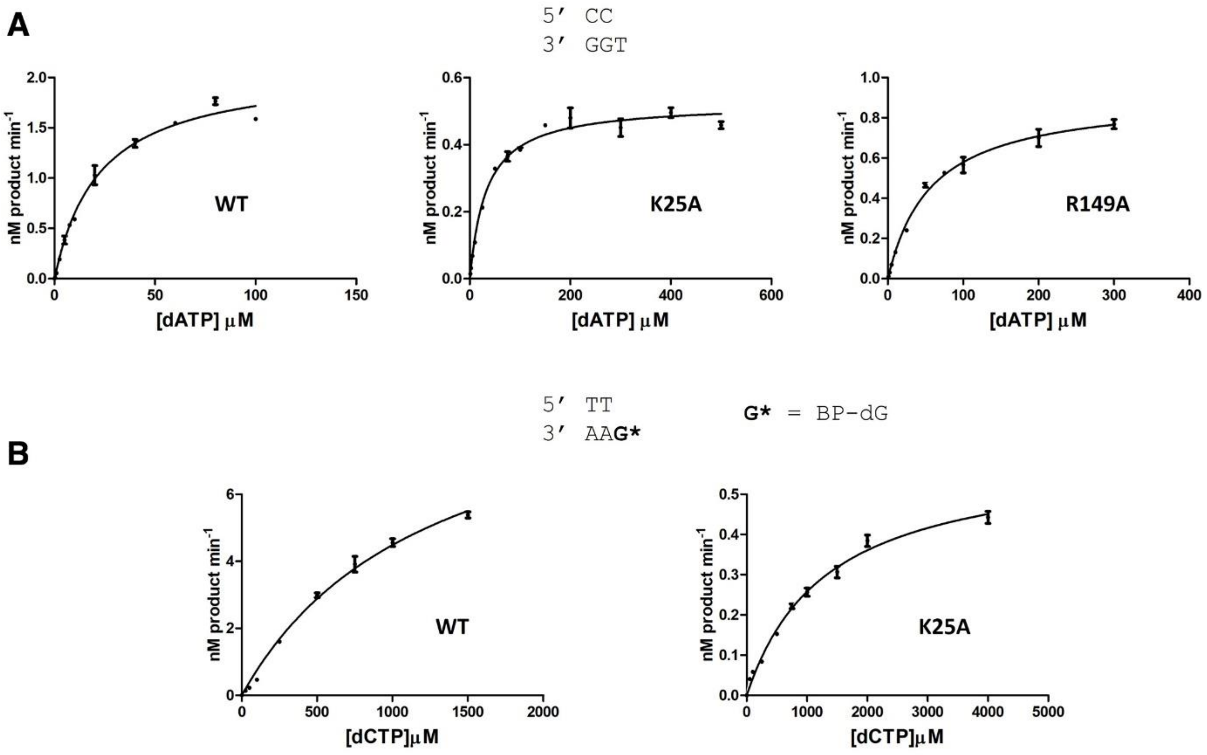

Figure S6. Kinetics of single nucleotide insertion opposite (A) undamaged T or (B) BP-dG template by WT and mutant polκ. The plots show rate of reaction as a function of dNTP concentration. The steady-state kinetics of single-nucleotide incorporation was conducted in a 10-μL reaction with 50 nM DNA substrates, (A) 0.1 nM of WT, 0.25 nM of K25A, or 0.1 nM of R149A (B) 5 nM WT or 25 nM K25A, and variable concentrations of a single nucleotide. Reaction times were varied to ensure that the maximal product formation was <20%. The reaction products were separated by electrophoresis on a 20% denaturing polyacrylamide gel. The percentage of primers extended was estimated from the phosphorimager densitometry scans and the observed rate of reaction was then plotted as a function of dNTP concentration. The parameters k_{cat} and K_m were estimated from the nonlinear regression fit of the Michaelis-Menten equation using GraphPad Prism 5 software. All experiments were carried out in triplicates. Error bars indicate the standard deviation.

```
EcKF     747 ETVTSEQRRSAKAINFGLIYGMSAFGLARQLNIPRKEAQKYMDLYFERYPGVLEYMERTR 806
BspolI   695 EDVTANMRRQAKAVNFGIVYGISDYGLAQNLNITRKEAAEFIERYFASFPGVKQYMDNIV 754
T7pol    511 IAAELPTRDNAKTFIYGFLYGAGDEKIGQIVGAGKERGKELKKKFLENTPAIAALRESIQ 570
TapolI   652 EAVDPLMRRAAKTINFGVLYGMSAHRLSQELAIPYEEAQAFIERYFQSFPKVRAWIEKTL 711
               .    *  **:. :*.:** .   :.: :    :..    . ::   * :    :
```

Figure S7. Amino acid sequence alignments of A-family DNA polymerases. *Ec*KF: *E coli* Klenow fragment; *Bs*pol I: *Bacillus stearothermophilus* pol I; T7pol: Bacteriophage T7 DNA polymerase; and Tapol I: *Thermus aquaticus* pol I. The conserved lysine residues shown in Figure 3 and the equivalent lysine in *Ec*KF are highlighted in yellow.

Table S1. The +1 charged residues at the equivalent position to K25 of polκ in DNA pols.

Enzyme	Family	Equivalent +1 residue
Bacillus stearothermophilus DNA pol I	A	K706
Thermus aquaticus pol I	A	K663
T7 DNA polymerase	A	K522
E. coli DNA Pol I: Klenow Fragment (KF)	A	K758
E. coli DNA Pol II	B	K493
Human DNA polymerase eta (polη)	Y	R61
HIV-1 reverse transcriptase (RT)	RT	R72
